# Seasonal human coronavirus humoral responses in AZD1222 (ChaAdOx1 nCoV-19) COVID-19 vaccinated adults reveal limited cross-immunity

**DOI:** 10.3389/fimmu.2024.1401728

**Published:** 2024-05-17

**Authors:** Ann Marie Stanley, Anastasia A. Aksyuk, Deidre Wilkins, Justin A. Green, Dongmei Lan, Kathryn Shoemaker, Hong-Van Tieu, Magdalena E. Sobieszczyk, Ann R. Falsey, Elizabeth J. Kelly

**Affiliations:** ^1^ Translational Medicine, Vaccines & Immune Therapies, BioPharmaceuticals R&D, AstraZeneca, Gaithersburg, MD, United States; ^2^ Clinical Development, Vaccines & Immune Therapies, BioPharmaceuticals R&D, AstraZeneca, Cambridge, United Kingdom; ^3^ Biometrics, Vaccines & Immune Therapies, BioPharmaceuticals R&D, AstraZeneca, Gaithersburg, MD, United States; ^4^ Division of Infectious Diseases, Department of Medicine, Vagelos College of Physicians and Surgeons, New York-Presbyterian Columbia University Irving Medical Center, New York, NY, United States; ^5^ Lindsley F. Kimball Research Institute, New York Blood Center, New York, NY, United States; ^6^ Division of Infectious Diseases, Department of Medicine, Vagelos College of Physicians and Surgeons, New York-Presbyterian/Columbia University Irving Medical Center, New York, NY, United States; ^7^ Department of Medicine, Infectious Diseases, University of Rochester School of Medicine and Dentistry, Rochester, NY, United States; ^8^ Infectious Disease, Rochester Regional Health, Rochester, NY, United States; ^9^ Formerly Translational Medicine, Vaccines & Immune Therapies, BioPharmaceuticals R&D, AstraZeneca, Gaithersburg, MD, United States

**Keywords:** AZD1222 (ChaAdOx1 nCoV-19), seasonal coronavirus, human coronaviruses, cross-immunity, SARS-CoV-2, COVID-19, humoral responses, COVID-19 vaccination

## Abstract

**Background:**

Immunity to severe acute respiratory syndrome coronavirus 2 (SARS-CoV-2) is now widespread; however, the degree of cross-immunity between SARS-CoV-2 and endemic, seasonal human coronaviruses (HCoVs) remains unclear.

**Methods:**

SARS-CoV-2 and HCoV cross-immunity was evaluated in adult participants enrolled in a US sub-study in the phase III, randomized controlled trial (NCT04516746) of AZD1222 (ChAdOx1 nCoV-19) primary-series vaccination for one-year. Anti-HCoV spike-binding antibodies against HCoV-229E, HCoV-HKU1, HCoV-OC43, and HCoV-NL63 were evaluated in participants following study dosing and, in the AZD1222 group, after a non-study third-dose booster. Timing of SARS-CoV-2 seroconversion (assessed via anti-nucleocapsid antibody levels) and incidence of COVID-19 were evaluated in those who received AZD1222 primary-series by baseline anti-HCoV titers.

**Results:**

We evaluated 2,020/21,634 participants in the AZD1222 group and 1,007/10,816 in the placebo group. At the one-year data cutoff (March 11, 2022) mean duration of follow up was 230.9 (SD: 106.36, range: 1–325) and 94.3 (74.12, 1–321) days for participants in the AZD1222 (n = 1,940) and placebo (n = 962) groups, respectively. We observed little elevation in anti-HCoV humoral titers post study-dosing or post-boosting, nor evidence of waning over time. The occurrence and timing of SARS-CoV-2 seroconversion and incidence of COVID-19 were not largely impacted by baseline anti-HCoV titers.

**Conclusion:**

We found limited evidence for cross-immunity between SARS-CoV-2 and HCoVs following AZD1222 primary series and booster vaccination. Susceptibility to future emergence of novel coronaviruses will likely persist despite a high prevalence of SARS-CoV-2 immunity in global populations.

## 1 Introduction

The use of coronavirus disease 2019 (COVID-19) vaccines following the global circulation of the novel betacoronavirus severe acute respiratory syndrome coronavirus 2 (SARS-CoV-2) has been instrumental to reducing the health burden of the COVID-19 pandemic (1, 2). SARS-CoV-2 immunity is now widespread due to COVID-19 vaccination and/or natural infection (3–6). However, despite notable population immunity, SARS-CoV-2 is endemic in many regions due to the continued emergence and circulation of immune evasive variants (3–5, 7, 8). Thus, mitigating the impact of both SARS-CoV-2 variants and future novel coronaviruses with pandemic potential is a global health imperative.

It is unclear to what degree SARS-CoV-2 immunity also provides cross-immunity against other pathogenic coronaviruses, or ‘universal coronavirus’ immunity. Elucidating the degree of cross-immunity between SARS-CoV-2 and other known coronaviruses has the potential to inform pan-coronavirus prevention strategies. AZD1222 (ChAdOx1 nCoV-19), a replication-deficient adenovirus-vectored COVID-19 vaccine generated against ancestral SARS-CoV-2 spike protein, is well characterized. The safety, immunogenicity, and efficacy of AZD1222 has been repeatedly demonstrated in several large clinical trials, including a randomized, placebo controlled, phase III study in the United States (US), Chile, and Peru (9–11). Additionally, AZD1222 is one of the most widely administered vaccines. More than 2 billion doses of AZD1222 were supplied to 170 countries within the first year of administration (12), and the World Health Organization continues to recommend AZD1222 for use in vaccination strategies aimed at maintaining SARS-CoV-2 immunity (13). AZD1222 is therefore an ideal model for evaluating the potential for cross-immunity between SARS-CoV-2 and other coronaviruses.

It is not currently feasible to identify zoonotic coronavirus lineages with the potential for extensive circulation in humans (14). However, there are four identified endemic human coronaviruses (HCoVs) that may offer insights into coronavirus cross-immunity after COVID-19 vaccination; the alphacoronaviruses HCoV-229E and HCoV-NL63, and the betacoronaviruses HCoV-OC43 and HCoV-HKU1 (15). HCoVs exhibit a high level of antigenic variation and cyclical circulation in global populations, including in the US (16). HCoV infections are most frequently observed in children and exhibit notable annual peaks during winter months, a characteristic leading to their designation of seasonal coronavirus (15–17). While severe illness is possible from HCoVs, particularly in elderly individuals who acquire HCoV-OC43 (18, 19), they have primarily been documented as the causative pathogen for mild respiratory illnesses in healthy adults and adolescents (17).

Since the emergence of SARS-CoV-2, several studies have investigated the potential for cross-immunity between HCoVs and SARS-CoV-2, with mixed results. Although the HCoV lineages are antigenically variable, they share some sequence homology with SARS-CoV coronaviruses, particularly the HCoV betacoronaviruses (20, 21). Additionally, the alphacoronavirus HCoV-NL63 relies on the same receptor for viral entry as SARS-CoV-2, angiotensin converting enzyme 2 (22). More tangibly, pre-existing humoral and cellular immune responses to SARS-CoV-2 were detected in pre-pandemic samples, suggesting that prior infection with HCoVs may impact SARS-CoV-2 immunity (23–26). Cross-reactive humoral responses have been suggested to occur after HCoV infection (27), SARS-CoV-2 infection, and COVID-19 vaccination (28–30), although the degree of cross-protection afforded is unclear. Additionally, surveillance data indicates that there was a substantial drop in seasonal coronavirus cases during the COVID-19 pandemic (16, 23) as well as decreased susceptibility to severe COVID-19 in children and adolescents, who have also been shown to have the highest HCoV infection rates (16, 17, 31, 32). However, both phenomena have been credibly attributed to alternative causes, such as non-pharmaceutical interventions (33) and differential innate immune responses in children (31).

Drawing conclusions on cross-immunity between HCoV lineages and SARS-CoV-2 has been hindered by the limitations of available HCoV immunity data, as data have been primarily collected either from small-scale clinical studies, or viral surveillance in select regions or populations (15, 17). Herein, we analyze humoral responses to HCoVs after COVID-19 vaccination in a cohort of sub-study participants enrolled in the US from a large-scale phase III clinical trial of two-dose AZD1222 primary series to explore potential cross-immunity between HCoVs and SARS-CoV-2.

## 2 Materials and methods

### 2.1 Study design and ethics

This work is based on a phase III, randomized, placebo-controlled trial (ClinicalTrials.gov number, NCT04516746) which was conducted at 88 sites in the US, Chile, and Peru. Full details of the trial design and participants, including primary safety, immunogenicity, and efficacy endpoints have been reported previously (9, 10). Eligible individuals included adults (aged ≥18 years) who were healthy, had medically stable chronic diseases, and an increased risk for SARS-CoV-2 infection. Individuals with a history of laboratory-confirmed SARS-CoV-2 infection, confirmed or suspected immunosuppressive/immunodeficiency status, or recurrent severe infections/use of immunosuppressant medication (excepting HIV-positive participants on stable antiretroviral therapy) were excluded. Participants were not evaluated for a history of HCoV infection at enrollment. Participants were randomized 2:1 to receive either AZD1222 (5 x 10^10^ viral particles) or saline placebo via intramuscular injection on study days 1 and 29. The analyses herein were restricted to participants enrolled in the US who were included in a prespecified sub-study that provided additional samples for the further evaluation of exploratory immunogenicity endpoints (9, 10, 34), through one year of follow-up (day 360; data cutoff, March 11, 2022).

This trial was conducted in compliance with the principles of the Declaration of Helsinki and the International Council for Harmonization Good Clinical Practice guidelines. The protocol and amendments for this trial (9, 10) were approved by the ethics committee or institutional review board at each center. Prior to enrolment all participants provided informed consent. During the study, participants were unblinded to facilitate the receipt of non-study COVID-19 vaccinations once available through emergency-use authorizations (EUA). The full details, the study timeline, and amendments to the study protocol are outlined in Sobieszczyk et al. (9).

### 2.2 Procedures

#### 2.2.1 Illness visits and COVID-19 case definition

Participants who presented with qualifying symptoms provided nasopharyngeal swabs at illness visits during the study, as previously described (34). Samples were tested for SARS-CoV-2 infection using reverse transcriptase polymerase chain react (RT-PCR) via validated assay performed at Labcorp (Burlington, NC, USA). COVID-19 cases were defined as either a positive SARS-CoV-2 PCR-result from testing conducted at a study illness visit or a documented event of COVID-19 occurring at least 15 days after receiving a second dose of the study intervention and prior to the receipt of a non-study COVID-19 vaccination.

#### 2.2.2 HCoV and RSV Biofire assay

Due to prespecified testing protocols, only participant nasopharyngeal swabs that tested negative for SARS-CoV-2 after illness visits were subsequently tested for 20 respiratory pathogens in a multiplexed nucleic acid amplification test which included HCoV and respiratory syncytial virus (RSV), a non-coronavirus seasonal respiratory virus which served as a negative control. Consequently, participants who may have been concurrently infected with SARS-CoV-2 and either HCoV or RSV were not detected, or included, in these analyses. Both HCoV and RSV were defined by positivity in a validated BioFire assay performed by Labcorp (Indianapolis, IN, USA) using the Biofire^®^ Respiratory Panel 2.0.

#### 2.2.3 Serum sample collection and processing

Serum samples were collected for HCoV serological assessments from sub-study participants at predetermined study visits on study days 1, 29, 57, 180, and 360 (pre-dose on days 1 and 29) (9, 10, 34). For SARS-CoV-2 serological assessments, samples were collected at two additional visits on days 15 and 43. Briefly, all collected serum samples were allowed to clot at room temperature, centrifuged within 1 hour of collection (before being stored at –70 °C), and shipped to specialized central laboratories for humoral immunogenicity analyses (34).

#### 2.2.4 Multiplex serology IgG assays

A qualified multiplex electrochemiluminescence (ECL) immunoassay (35) developed at Meso Scale Diagnostics (Rockville, MD, USA) and performed and validated at PPD Vaccines (Richmond, VA, USA), was used to quantitate serum immunoglobulin G levels of anti-SARS-CoV-2 and anti-HCoV (HCoV-229E, HCoV-HKU1, HCoV-NL63, and HCoV-OC43) spike-binding antibodies, as well as anti-RSV post-fusion F (post-F) protein antibodies in a similar manner as described previously (9, 10, 34, 35). Briefly, a Meso Scale Discovery^®^ (Rockville, MD, USA) platform was used to determine antibody concentrations through indirect binding. ECL signal outputs were interpolated relative to a standard curve generated from serially diluted reference standards (comprised of pooled human serum samples). Concentrations are reported as geometric mean titers (the antilogarithm of Σ(log2 transformed titer/number of participants) as AU/mL is an arbitrary concentration determined from reference standards. For anti-HCoV spike-binding antibodies and anti-RSV post-F antibody levels, reference standards were comprised of pooled human serum containing antibodies specific for the specified HCoV lineage spike and RSV post-F protein antigens.

#### 2.2.5 SARS-CoV-2 nucleocapsid seroconversion

Anti-SARS-CoV-2 nucleocapsid assessments were performed via validated assay by PPD Vaccines (Richmond, VA, USA), as previously described (35). A post-treatment SARS-CoV-2 seroconversion event was defined as the first incidence of nucleocapsid antibody assay titer above a previously defined threshold (>9787 AU mL^-1^) (35) occurring at least 15 days following receipt of a second dose.

#### 2.2.6 SARS-CoV-2 pseudovirus neutralizing assay

SARS-CoV-2 pseudovirus neutralizing antibody (nAb) levels were determined via a validated lentivirus-based ancestral (Wuhan D614) assay by Labcorp-Monogram Biosciences (South San Francisco, CA, USA) (9, 10, 34). Briefly, HIV-1 pseudovirions that express the SARS-CoV-2 spike protein were incubated with serial dilutions of participant serum. Titers were measured by assessing the inhibition of luciferase activity in target cells expressing the angiotensin-converting enzyme-2 receptor after exposure to the pseudovirions. The presence of anti-SARS-CoV-2 nAbs in serum inhibits the infection of, and subsequent expression of luciferase activity in, target cells; therefore, titers are reported as the reciprocal of the serum dilution conferring 50% inhibition (ID_50_) of pseudovirus infection, as follows:


$$\begin{array}{l} \%\text{ inhibition }=\;100\%\;–\;\left(\left(\left(\text{RLU}\left(\text{Vector}+\text{Sample}+\text{Diluent}\right)\right.\;–\text{ RLU}(\text{Background}\right.\right)/\\ \;\left.\left.\left(\text{RLU}\left(\text{Vector}+\text{Diluent}\right)\;–\text{ RLU}\left(\text{Background}\right)\right)\right)\;\times \;100\%\right) \end{array}$$

### 2.3 Analysis populations

The analyses in this manuscript are restricted to participants in the US enrolled sub-study population, as previously detailed (9, 10, 34). All participants were censored at the date of non-study COVID-19 vaccination, or the last trial contact prior to the data cutoff, excepting in analyses investigating humoral immunogenicity after non-study vaccination. For analyses of post-primary series immunogenicity, data points after dosing (i.e., post-study day 29) were restricted to participants who had received two doses of AZD1222 and had remained in the study for at least 15 days following dosing.

### 2.4 Statistics

For ethical reasons, study participants were unblinded and notified once they qualified for an EUA and/or approved non-study COVID-19 vaccination. The censoring implications of this decision are fully detailed by Sobieszczyk et al. (9). In September 2021, the EUA for monovalent mRNA vaccinations was modified to include third-dose boosters in the US (36, 37). Participants were censored in all immunogenicity analyses at the date of non-study COVID-19 vaccinations unless otherwise specified.

The sub-study population for the evaluation of exploratory immunogenicity endpoints and included the first approximately 3,000 participants randomized in the US and was intended to comprise 1,500 participants aged 18–55 years, 750 aged 56–69 years, and 750 aged ≥70 years. The analyses herein employed descriptive statistics, including: geometric mean titers, the number of participants, first and third titer quartiles, interquartile range (IQR), and median titer values titer values.

## 3 Results

### 3.1 Study design and participants

Out of 32,450 participants enrolled in the phase III study of primary-series AZD1222 between August 28, 2020 and January 15, 2021 underwent randomization (2:1) to receive either AZD1222 (n = 21,634) or placebo (n = 10,816), as described previously (9). The analyses herein are restricted to a sub-study population of 2,030 participants in the AZD1222 group and 1,012 in the placebo group comprised from the first participants to enroll at the US study sites. Data cutoff for these analyses was March 11, 2022, encompassing a follow-up duration of approximately one year (through study day 360).

A total of 2,026 AZD1222 group and 1,009 placebo group sub-study participants provided serological samples for exploratory immunogenicity analyses. Baseline characteristics were balanced between these groups (**Supplementary Table 1**).

Immunogenicity against HCoVs was evaluated over the course of one year in 2,020 participants in the AZD1222 group and 1,007 participants in the placebo group. Of those, 1,940 and 962, respectively, remained in the sub-study for at least 15 days following completion of dosing. Mean duration of follow-up in these participants was 230.9 (SD: 106.36, range: 1–325) and 94.3 (74.12, 1–321) days, respectively. A total of 754 sub-study participants who completed a AZD1222 primary series and subsequently reported the receipt of a non-study COVID-19 vaccine (third-dose booster) by day 360 were evaluated for anti-HCoV humoral responses following a booster dose.

### 3.2 HCoV immunogenicity over time in sub-study participants

**Figure 1:**
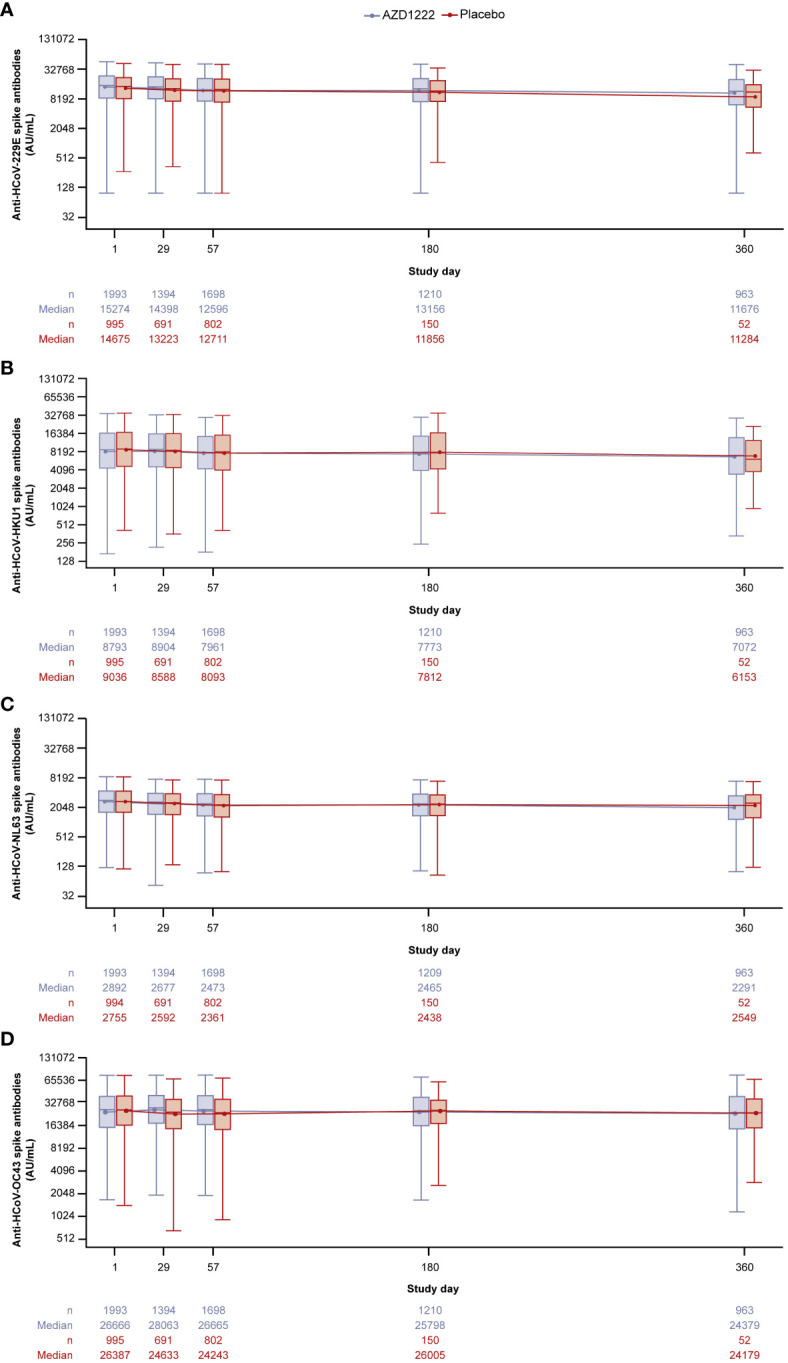
Anti-HCoV spike-binding antibody titers in participants over time for **(A)** HCoV-229E **(B)** HCoV-HKU1 **(C)** HCoV-NL63 **(D)** HCoV-OC43. Participants were censored on the date of the first reported instance of non-study COVID-19 vaccination. The bottom and top edges of the box indicate the first and third quartiles (the difference is the IQR) and the line inside the box is the median. The line connects the geometric mean value for every visit. Whiskers extending up to 1.5 times the IQR above the upper quartile and below the lower quartile. Any points more than 1.5 x IQR from the box were considered outliers and are not presented. The boxplots are presented in log2 scale. Baseline is defined as the last non-missing measurement taken prior to the first dose of study intervention (including unscheduled measurements, if any). Titer values measured as below LLoQ are imputed to half the LLoQ. Titer values measured as above ULoQ are imputed at the ULoQ value. Assessments collected after non-study COVID-19 vaccine administration/exclusionary restricted medication intake are excluded, regardless of unblinding. HCoV, human coronavirus, IQR, interquartile range, LLoQ, lower limit of quantification, ULoQ, upper limit of quantification.

Serum IgG anti-HCoV spike-binding antibody levels for each HCoV lineage (HCoV-229E, HCoV-HKU1, HCoV-NL63, HCoV-OC43) were evaluated over time (**Figure 1**). Anti-HCoV geometric mean humoral responses were similar in participants who received AZD1222 or placebo and remained consistent. No notable elevations in anti-HCoV titers were observed after dosing (occurring on study days 1 and 29), nor was evidence of pronounced waning detected during follow up. Serum levels of anti-RSV post-F protein antibodies, a non-coronavirus respiratory virus which served as a negative control, also displayed no humoral response elevations after dosing as well as little evidence of waning (**Supplementary Figure 1**).

Booster doses of COVID-19 vaccines have been shown to further increase in humoral responses against SARS-CoV-2 compared to primary series (38). In order to determine if this increased magnitude of anti-SARS-CoV-2 humoral responses corresponded to changes in anti-HCoV humoral responses, we examined HCoV and SARS-CoV-2 immunity in AZD1222 group participants who had received a booster dose versus those who had not (**Supplementary Figure 2**). As expected, participants in the AZD1222 group who received a third-dose booster had higher SARS-CoV-2 nAb titers. In contrast, participants who received a booster dose did not display higher levels of, or a different pattern in, anti-HCoV antibody titers over time. Responses were consistent within each HCoV lineage regardless of whether participants received a booster vaccination by day 180, or between days 181 and 360 (**Supplementary Figure 3**). Anti-RSV post-F antibodies were also consistent overtime in both boosted and non-boosted participants (**Supplementary Figure 4**).

### 3.3 Baseline HCoV immunity and post-AZD1222 SARS-CoV-2 seroconversion

**Figure 2:**
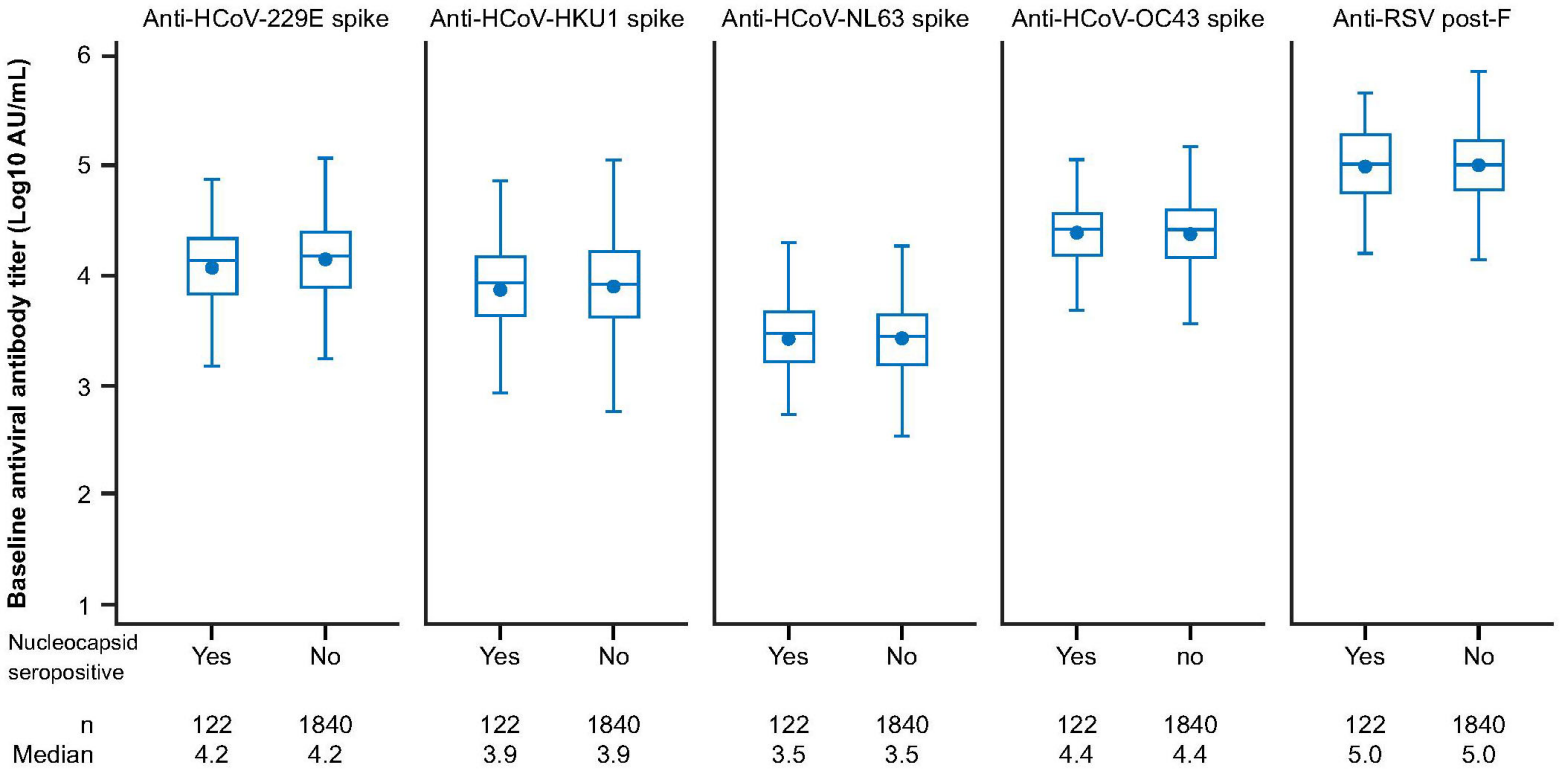
Anti-HCoV spike-binding antibody titers at baseline in sub-study participants who received two doses of AZD1222, by post-treatment SARS-CoV-2 nucleocapsid serostatus. Post-treatment SARS-CoV-2 seropositivity was defined by the presence of an above threshold (>9787 AU mL-1) (35) response to SARS-CoV-2 nucleocapsid antibodies occurring ≥15 days after receiving a second dose of AZD1222. The bottom and top edges of the box indicate the first and third quartiles (the difference is the IQR), the line inside the box is the median, and the marker inside the box is the geometric mean. Any points more than 1.5 x IQR from the box were considered outliers and are not displayed. The whiskers that extend from the box indicate the minimum and maximum after removing the outliers. Boxplots are created using log transformed values. Titer values measured as below LLoQ are imputed to half the LLoQ. Titer values measured as above ULoQ are imputed at the ULoQ value. HCoV, human coronavirus, IQR, interquartile range, LLoQ, lower limit of quantification, ULoQ, upper limit of quantification, SARS-CoV-2, severe acute respiratory syndrome coronavirus 2.

**Figure 3:**
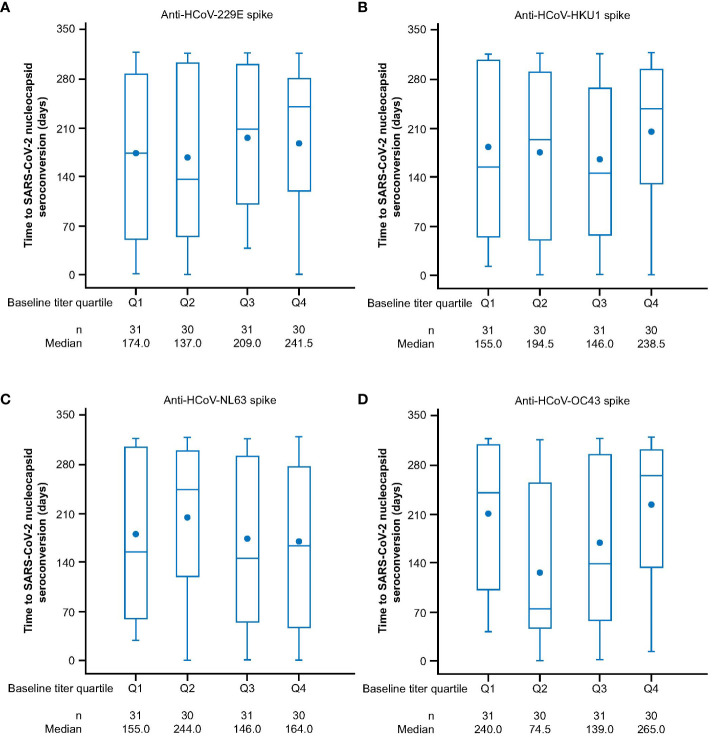
Time to SARS-CoV-2 nucleocapsid seroconversion in post-treatment SARS-CoV-2 nucleocapsid seropositive sub-study participants who received two doses of AZD1222, by HCoV titer quartile at baseline for **(A)** HCoV-229E **(B)** HCoV-HKU1 **(C)** HCoV-NL63 **(D)** HCoV-OC43. Participants who received two doses of AZD1222 were sorted by HCoV titer at baseline with quartile one denoting participants with the lowest baseline titers and quartile four the highest baseline anti-HCoV spike-binding antibody titers. Post-treatment SARS-CoV-2 seropositivity was defined by the presence of an above threshold (>9787 AU mL^-1^) (35) response to SARS-CoV-2 nucleocapsid antibodies occurring ≥15 days after receiving a second dose of AZD1222. The time to first post-treatment response has been calculated as follows: In days, the date of post-treatment response – (date of second dose of study intervention + 14) +1. For censored participants, the censoring time is from date of second dose of study intervention + 14, to last observed time during the analysis period/non-study COVID-19 vaccine administration. The bottom and top edges of the box indicate the first and third quartiles (the difference is the IQR), the line inside the box is the median, and the marker inside the box is the mean. Any points more than 1.5 x IQR from the box are considered outliers and are not displayed. The whiskers that extend from the box indicate the minimum and maximum after removing the outliers. Titer values measured as below LLoQ are imputed to half the LLoQ. Titer values measured as above ULoQ are imputed at the ULoQ value. HCoV, human coronavirus, IQR, interquartile range, LLoQ, lower limit of quantification, ULoQ, upper limit of quantification, SARS-CoV-2, severe acute respiratory syndrome coronavirus 2.

We next sought to investigate if prior exposure to HCoVs affects the dynamics of SARS-CoV-2 seroconversion by evaluating whether having higher baseline HCoV immunity correlated with delayed or reduced SARS-CoV-2 seroconversion. We analyzed 1,962 AZD1222 group participants who had received two doses of study intervention and from whom we had recorded baseline HCoV antibody titers to determine whether they experienced a SARS-CoV-2 seroconversion event (defined as having an above threshold, >9787 AU mL^-1^ (35), anti-SARS-CoV-2 nucleocapsid antibody titer occurring at least 15 days after receiving a second dose) during the follow-up period. We found that similar baseline titer levels were recorded in seropositive (n = 122, 6.2%) and seronegative (n = 1,840, 93.8%) participants who received an AZD1222 primary series across all HCoV linages (**Figure 2**). Additionally, when seropositive participants in the AZD1222 group were sorted into baseline titer quartiles for each HCoV lineage, there were no differences evident in the time to the first recorded SARS-CoV-2 seroconversion event (**Figure 3**).

### 3.4 COVID-19 cases over time by HCoV baseline titer levels

**Figure 4:**
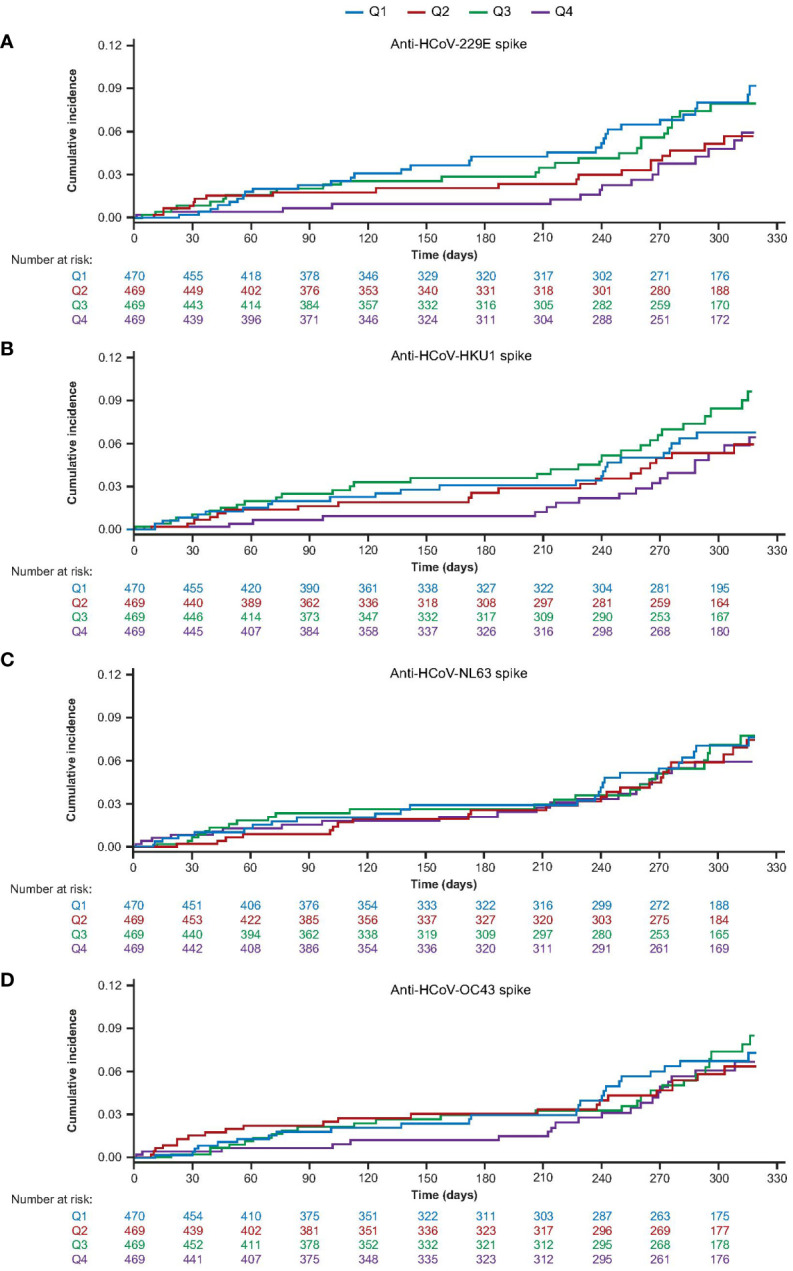
Cumulative incidence of post-treatment COVID-19 cases over time sub-study participants who received two doses of AZD1222, by baseline HCoV titer quartile for **(A)** HCoV-229E **(B)** HCoV-HKU1 **(C)** HCoV-NL63 **(D)** HCoV-OC43. Participants who received two doses of AZD1222 were sorted by HCoV titer at baseline with quartile one representing participants with the lowest baseline titers and quartile four the highest baseline anti-HCoV spike-binding antibody titers. Post-treatment COVID-19 cases were defined as those reported to occur ≥15 days after receiving a second dose of AZD1222. COVID-19 reported cases include any RT-PCR positive result or COVID-19 AEs, whichever occurred first. The time to first reported COVID-19 case was calculated as follows: in days, the date of reported COVID-19 – (date of receiving a second dose of study intervention + 14) + 1. For censored participants, the censoring time was defined as the date of second dose of study intervention + 14 up to the last study contact with the participant prior to the data cut-off/non-study COVID-19 vaccine administration. Participants who received a non-study COVID-19 vaccination prior to 15 days post second dose, or who did not have a baseline HCoV titer result, were excluded. The cumulative incidence curves are truncated at the point when less than 10% of participants remain at risk and active in the study, for each group. AE, adverse event, HCoV, human coronavirus, RT-PCR, reverse transcriptase polymerase chain reaction.

A total of 92 (4.9%) COVID-19 cases were detected in the AZD1222 group (**Supplementary Table 2**). To determine if COVID-19 cases may have been affected by HCoV immunity, we examined the incidence of post-treatment (at least 15 days after receiving the second dose of AZD1222) COVID-19 cases in participants sorted by baseline HCoV titer response quartile. Participants with the highest baseline levels of HCoV-229E were observed to exhibit numerically lower cumulative COVID-19 case incidence over time; participants in other baseline HCoV-229E quartiles had no detectable pattern in cumulative case incidence (**Figure 4A**). There were also no consistent patterns in cumulative COVID-19 case incidence for HCoV-HKU1, HCoV-NL63, or HCoV-OC43 lineages (**Figures 4B–D**).

An analysis of HCoV and RSV cases was not feasible as there were too few cases of seasonal coronavirus/HCoV (n = 13, 0.7%) and RSV (n = 4, 0.2%) identified in sub-study AZD1222 group participants to explore potential cross-protection after AZD1222 administration (**Supplementary Table 2**).

## 4 Discussion

Recently, the repeated emergence of novel coronaviruses with pandemic potential (e.g., SARS-CoV-1, SARS-CoV-2, and MERS-CoV) has highlighted the importance of reducing the risk of future coronavirus circulation to safeguard global health (14). However, it is unclear if maintaining high levels of SARS-CoV-2 immunity, through COVID-19 vaccination and/or natural infection, has the potential to lead to ‘universal coronavirus’ immunity. To our knowledge, the findings herein represent the largest cohort of participants in which long-term humoral antibody responses against HCoVs have been examined, including after both AZD1222 primary-series and heterologous booster vaccination. We found that there was little change in HCoV humoral responses over the course of one year, as reflected by consistent levels of anti-HCoV spike-binding antibody levels in both AZD1222 and placebo groups. Additionally, we found that patterns in anti-HCoV humoral responses were remarkably consistent with anti-RSV humoral responses, a non-coronavirus respiratory virus which served as a negative control in this study. Anti-HCoV humoral responses remained consistent even after participants received booster vaccinations, despite notable elevation in SARS-CoV-2 pseudovirus neutralizing antibodies, further suggesting that COVID-19 vaccination had limited effects on anti-HCoV humoral responses. Taken together we find limited evidence that vaccination with AZD1222 primary series, or the receipt of a third-dose booster, influences humoral immunity against HCoVs.

It has also been theorized that HCoV infection prior to COVID-19 vaccination primes immune responses against SARS-CoV-2, thereby increasing post-vaccination immunity and potentially reducing COVID-19 severity (28, 29, 31). Our findings suggest that higher baseline anti-HCoV spike-binding antibody levels did not, in general, correlate with delayed or reduced SARS-CoV-2 seroconversion. However, participants with the highest baseline titers against HCoV-229E also had the lowest cumulative incidence rate of reported COVID-19 cases over time. The differences observed may indicate that cross-immunity between HCoV-229E and SARS-CoV-2 has some limited effects on SARS-CoV-2 infection/severity (reported COVID-19 cases were largely symptomatic). This finding is inconsistent with our observations that there were no obvious trends between baseline HCoV-229E anti-spike binding antibody levels and SARS-CoV-2 seroconversion post-AZD1222, but cross-coronavirus immune dynamics could be mediated by mechanisms that were not examined in this study, such as HCoV neutralizing antibodies (39) or T-cell mediated immune responses (24). However, we are also unable to rule out that the finding was coincidental, especially given the absence of a discernible pattern in other HCoV-229E baseline response quartiles as well as the low level of spike homology between HCoV-229E and SARS-CoV-2.

The frequency of HCoV infections and other respiratory viral infections, including RSV, decreased in US during the 2020–2021 respiratory season, a period concurrent with data collection (16, 40). Therefore, this study also presented a unique opportunity to examine the durability of infection-induced immunity to HCoVs. Unexpectedly, we observed that there were remarkably consistent humoral responses over time in sub-study participants (from baseline to one year) for all HCoVs, despite detecting very few HCoV cases during the study. Our finding of limited waning is especially interesting considering evidence in pre-pandemic studies that, despite normally high HCoV seroprevalence in global populations, HCoV symptomatic reinfection occurs readily at 12 months (15, 41). This, coupled with evidence of durable HCoV humoral responses indicates that HCoV reinfection could occur despite high average HCoV humoral immunity in a population. If so, this suggests that future susceptibility to emergent SARS-CoV-2 variants and novel coronaviruses may continue to persist even if SARS-CoV-2 immunity is maintained in global populations.

While HCoVs offer an ideal model to studying cross-coronavirus immunity due to their high level of antigenic variation and robust co-circulation in populations (16, 20), the contradictory findings above further highlight the need for more research into HCoV immune dynamics and protection to better inform design and interpretation of studies exploring pan-coronaviral immunity. Indeed, there is some evidence that the cross-immunity between HCoVs and SARS-CoV-2 immunity may be highly complex and dependent on several factors, including high variability between adolescents and adults (30, 32, 42). As these results were restricted to adult participants, an analysis of HCoV immunity in adolescents was not feasible for this study. Nevertheless, when taken together, our results suggest that pre-existing HCoV immunity has limited effects on cross-protection against SARS-CoV-2 following AZD1222 vaccination, a finding that is consistent with other studies following COVID-19 vaccination (43, 44).

In contrast to our findings of limited waning of anti-HCoV humoral responses, the kinetics of SARS-CoV-2 humoral immunity after either vaccination, or natural immunity have been shown to wane within one year (38, 45). The differences in the durability of humoral responses against HCoV and SARS-CoV-2 suggest two possible interpretations: 1) Repeated annual exposure may provoke longer duration of elevated antibody responses against HCoVs than we have observed with SARS-CoV-2. An immune dynamic that would also be consistent with our, and others, findings of a slow decline in anti-RSV humoral responses, another seasonal virus associated with frequent reinfection (46). If this is true, a trend towards more durable anti-SARS-CoV-2 immune responses may be observed in the future, perhaps consistent with differences already observed in humoral response kinetics after hybrid immunity (6) or elongated dosing schedules (47). 2) SARS-CoV-2 immunity may be less durable than immunity to HCoVs, and potentially other coronaviruses, limiting the potential for maintaining long-term coronavirus cross-immunity from vaccination. Studies in SARS-CoV-1, SARS 2003 pandemic pathogen and the most closely related coronavirus to SARS-CoV-2 with human circulation, have found humoral responses persisted for an average of two years after infection (48, 49), and anti-MERS-CoV humoral responses detectable in most survivors after one year (15), lending credence to the suggestion of divergent duration of SARS-CoV-2 immune responses. However, studies have also shown that SARS-CoV-1 survivors exhibited increased humoral immunity to SARS-CoV-2 after COVID-19 vaccination (50, 51) and past symptomatic MERS-CoV infection was shown to reduce the risk of COVID-19 disease (52), indicating that cross-coronavirus immunity may be readily provoked after detectable humoral immunity wanes.

Limitations for this study included the preclusion of an assessment of protection against seasonal coronavirus after AZD1222 due to the low event rate of confirmed HCoV infection, likely due to a concurrent reduction in US respiratory infections (16, 39, 53). Also, participants who had co-infections of SARS-CoV-2 and HCoV/RSV were not included in these analyses due to the sequence of testing employed; although, we would expect such cases to be rare. Due to the above limitations, it was not possible to determine the magnitude of immune responses promoted immediately after infection with HCoVs or RSV. In other words − to what extent are the observed responses reflective of already waned immunity in the participants? This illustrates why timing of data collection is important to consider when examining the immune dynamics of seasonal infections, especially as the robust resumption of HCoV circulation since the conclusion of this study tentatively supports our findings of limited cross-protection between SARS-CoV-2 and HCoVs (54). An additional limitation of our study was that the analyses were restricted to anti-HCoV spike-binding rather than neutralizing antibodies. Both HCoV spike-binding and neutralizing antibodies have historically displayed low cross-reactivity between lineages, with minimal overlap of epitopes/cross-reactivity, particularly between alphacoronaviruses and betacoronaviruses (15). However, we cannot rule out the possibility of greater induction of post-vaccination HCoV neutralizing antibody responses than spike-binding antibodies. Furthermore, T-cell mediated immunity is known to be a key contributing factor in protection against, or severity of, COVID-19 disease and both natural SARS-CoV-2 infection and COVID-19 vaccination have been shown to elicit robust T-cell responses (55–57). Taken together with evidence that cross-reactive, mostly memory, CD4^+^ T cells, with affinity to both SARS-CoV-2 and HCoVs were detectible in pre-pandemic blood samples, it is likely that coronaviral cross-immunity may also be mediated by T cells (24). Lastly, a full assessment of SARS-CoV-1 and MERS-CoV immunity was beyond the scope of this study, precluding a complete assessment of pan-coronavirus immunity following AZD1222.

In conclusion, in this one-year analysis of endemic HCoV immunity in sub-study participants enrolled in a large-scale clinical trial of AZD1222 primary series, we found limited evidence for cross-immunity against HCoVs following either AZD1222, or third dose booster vaccination. These findings support the prediction that susceptibility to future emergence of novel coronaviruses will persist despite a high predominance of SARS-CoV-2 seropositivity and/or COVID-19 vaccination in global populations. They also highlight that there is an unmet need for a more complete understanding of HCoV immune dynamics to inform future pan-coronavirus prevention strategies.

## Data availability statement

The datasets presented in this article are not readily available because the data underlying the findings described in this article may instead be obtained in accordance with AstraZeneca’s data sharing policy described at https://astrazenecagrouptrials.pharmacm.com/ST/Submission/Disclosure. Data for studies directly listed on Vivli can be requested through Vivli at www.vivli.org. Data for studies not listed on Vivli could be requested through Vivli at https://vivli.org/members/enquiries-about-studies-not-listed-on-the-vivli-platform/. AstraZeneca Vivli member page is also available outlining further details: https://vivli.org/ourmember/astrazeneca/. Requests to access the datasets should be directed to https://astrazenecagrouptrials.pharmacm.com/ST/Submission/Disclosure.

## Ethics statement

The studies involving humans were approved by the ethics committee or institutional review board at each center. The protocol and amendments for this trial (ClinicalTrials.gov NCT04516746) were therefore approved by the ethics committee or imitational review board at each center and the trial was conducted in compliance with the principles of the Declaration of Helsinki and the International Council for Harmonization Good Clinical Practice guidelines. The studies were conducted in accordance with the local legislation and institutional requirements. The participants provided their written informed consent to participate in this study.

## Author contributions

AS: Conceptualization, Formal Analysis, Investigation, Methodology, Project administration, Visualization, Writing – original draft, Writing – review & editing. AA: Conceptualization, Data curation, Methodology, Writing – original draft, Writing – review & editing. DW: Data curation, Methodology, Writing – original draft, Writing – review & editing. JG: Investigation, Project administration, Supervision, Writing – original draft, Writing – review & editing. DL: Formal Analysis, Methodology, Software, Supervision, Validation, Visualization, Writing – original draft, Writing – review & editing. KS: Formal Analysis, Software, Supervision, Validation, Visualization, Writing – original draft, Writing – review & editing. H-VT: Writing – original draft, Writing – review & editing, Data Curation. MS: Conceptualization, Data curation, Investigation, Writing – original draft, Writing – review & editing. AF: Methodology, Investigation, Data curation, Writing – original draft, Writing – review & editing. EK: Writing – original draft, Writing – review & editing, Conceptualization, Funding acquisition, Investigation, Methodology, Project administration, Resources, Supervision.
