# Supplementary material for: Seasonal human coronavirus humoral responses in AZD1222 (ChaAdOx1 nCoV-19) COVID-19 vaccinated adults reveal limited cross-immunity
Source: Front Immunol. 2024 May 17;15:1401728. doi: 10.3389/fimmu.2024.1401728 (PMC11143795; doi:10.3389/fimmu.2024.1401728)
Supplement: Supplementary file 1 [file DataSheet_1.docx]

Supplementary Material

Seasonal coronavirus (HCoV) humoral responses in AZD1222 (ChaAdOx1 nCoV-19) COVID-19 vaccinated adults reveal limited cross-immunity

**Ann Marie Stanley*, Anastasia A. Aksyuk, Deidre Wilkins, Justin A. Green, Dongmei Lan, Kathryn Shoemaker, Hong-Van Tieu, Magdalena E. Sobieszczyk, Ann R. Falsey, Elizabeth J. Kelly**

*** Correspondence:** Ann Marie Stanley; 1 Medimmune Way, Translational Medicine, Vaccines & Immune Therapies, BioPharmaceuticals R&D, AstraZeneca, Gaithersburg, MD, United States 20878; [annmarie.stanley@astrazeneca.com](mailto:annmarie.stanley@astrazeneca.com)


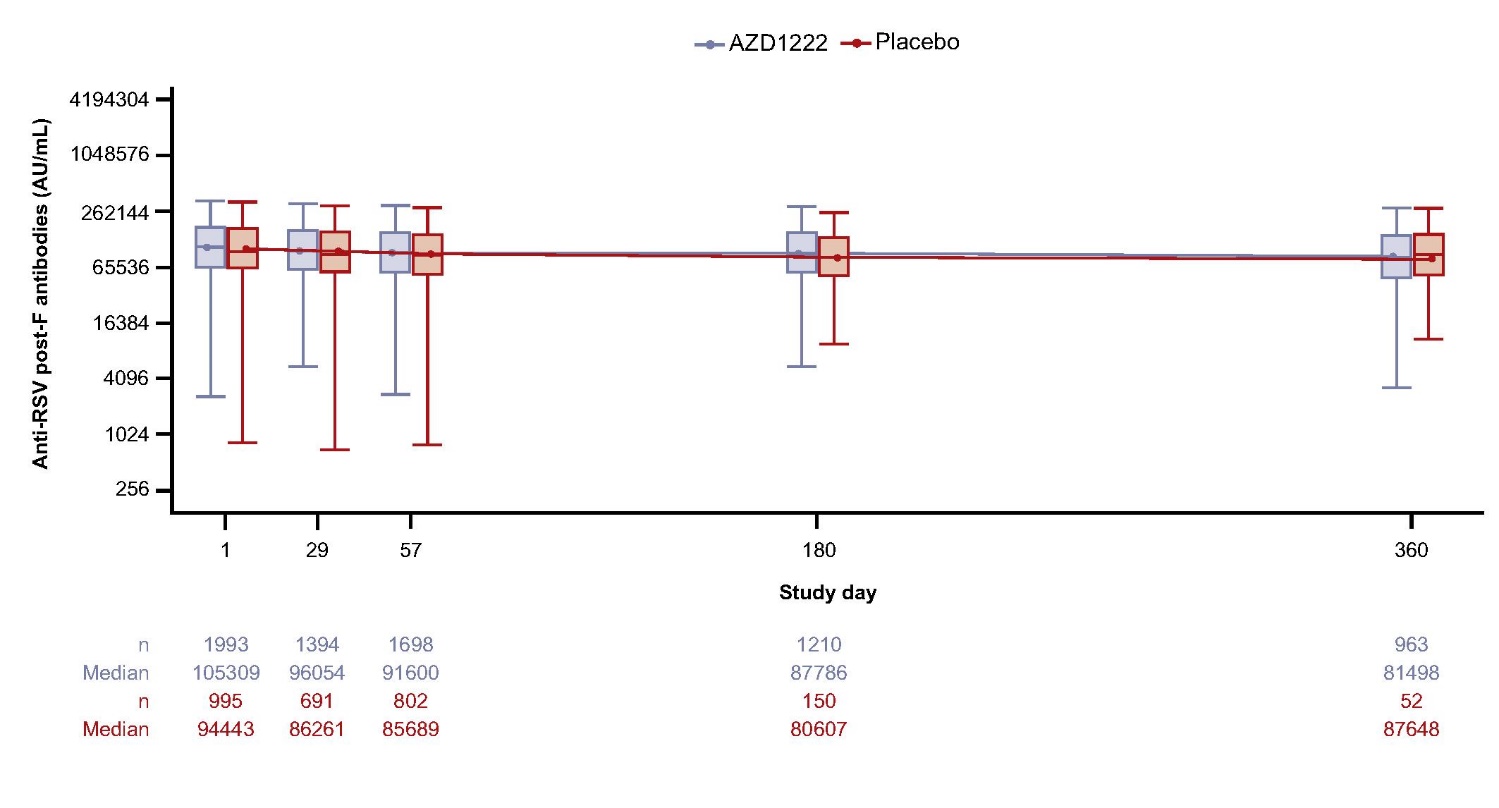
**Supplementary Figure 1.** **Anti-RSV post-F antibody titers in participants over time.** Participants were censored on the date of the first reported instance of non-study COVID-19 vaccination. The bottom and top edges of the box indicate the first and third quartiles (the difference is the IQR) and the line inside the box is the median. The line connects the geometric mean value for every visit. Whiskers extending up to 1.5 times the IQR above the upper quartile and below the lower quartile. Any points more than 1.5 x IQR from the box are considered outliers and are not presented. The boxplots are presented in log2 scale. Baseline is defined as the last non-missing measurement taken prior to the first dose of study intervention (including unscheduled measurements, if any). Titer values measured as below LLoQ are imputed to half the LLoQ. Titer values measured as above ULoQ are imputed at the ULoQ value. Assessments collected after non-study COVID-19 vaccine administration/exclusionary restricted medication intake are excluded, regardless of unblinding. IQR, interquartile range, LLoQ, Lower limit of quantification, post-F, post-fusion F, RSV, respiratory syncytial virus, ULoQ, upper limit of quantification.


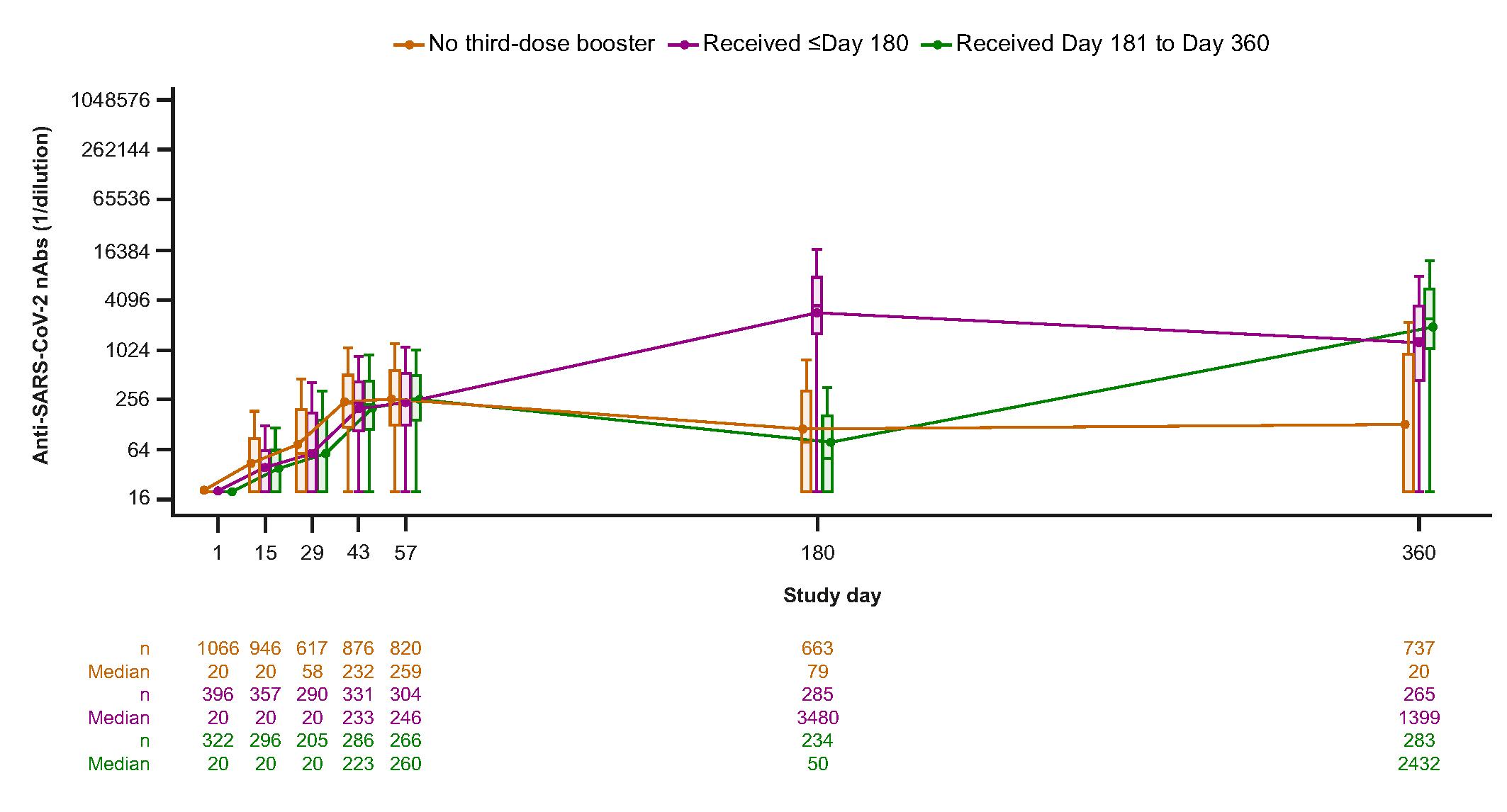
**Supplementary Figure 2. Ancestral SARS-CoV-2 neutralizing antibody titers over time in participants who received two doses of AZD1222, by receipt of a third-dose booster.** The bottom and top edges of the box indicate the first and third quartiles (the difference is the IQR) and the line inside the box is the median. The line connects the geometric mean value for every visit. Whiskers extending up to 1.5 times the IQR above the upper quartile and below the lower quartile. Any points more than 1.5 x IQR from the box are considered outliers and are not presented. The boxplots are presented in log2 scale. Baseline is defined as the last non-missing measurement taken prior to the first dose of study intervention (including unscheduled measurements, if any). Titer values measured as below LLoQ are imputed to half the LLoQ. Titer values measured as above ULoQ are imputed at the ULoQ value. Assessments collected after exclusionary restricted medication intake are excluded. The value at Day 1 is the last non-missing value taken prior to the first dose. Among sub-study participants evaluated for SARS-CoV-2 nAbs in the AZD1222 arm, n = 1091 had not received a third-dose booster through Day 360, n = 415 received a booster dose on or before Day 180 and n = 331 received a booster dose between Day 181 and Day 360. Participants who received a booster dose after Day 360 were not included in this analysis. IQR, interquartile range, LLoQ, lower limit of quantification, ULoQ, upper limit of quantification, SARS-CoV-2, severe acute respiratory syndrome coronavirus 2.

**Supplementary Figure 3. Anti-HCoV spike-binding titers over time in participants who received two doses of AZD1222, by receipt of a third-dose booster for** **(A)** HCoV-229E **(B)** HCoV-HKU1 **(C)** HCoV-NL63 **(D)** HCoV-OC43. The bottom and top edges of the box indicate the first and third quartiles (the difference is the IQR) and the line inside the box is the median. The line connects the geometric mean value for every visit. Whiskers extending up to 1.5 times the IQR above the upper quartile and below the lower quartile. Any points more than 1.5 x IQR from the box are considered outliers and are not presented. The boxplots are presented in log2 scale. Baseline is defined as the last non-missing measurement taken prior to the first dose of study intervention (including unscheduled measurements, if any). Titer values measured as below LLoQ are imputed to half the LLoQ. Titer values measured as above ULoQ are imputed at the ULoQ value. Assessments collected after exclusionary restricted medication intake are excluded. The value at Day 1 is the last non-missing value taken prior to the first dose. Among sub-study participants evaluated for anti-HCoV spike-binding titers in the AZD1222 arm, n = 1100 had not received a third-dose booster through Day 360, n = 419 received a booster dose on or before Day 180 and n = 335 received a booster dose between Day 181 and Day 360. Participants who received a booster dose after Day 360 were not included in this analysis. HCoV, human coronavirus; IQR, interquartile range, LLoQ, lower limit of quantification, ULoQ, upper limit of quantification.


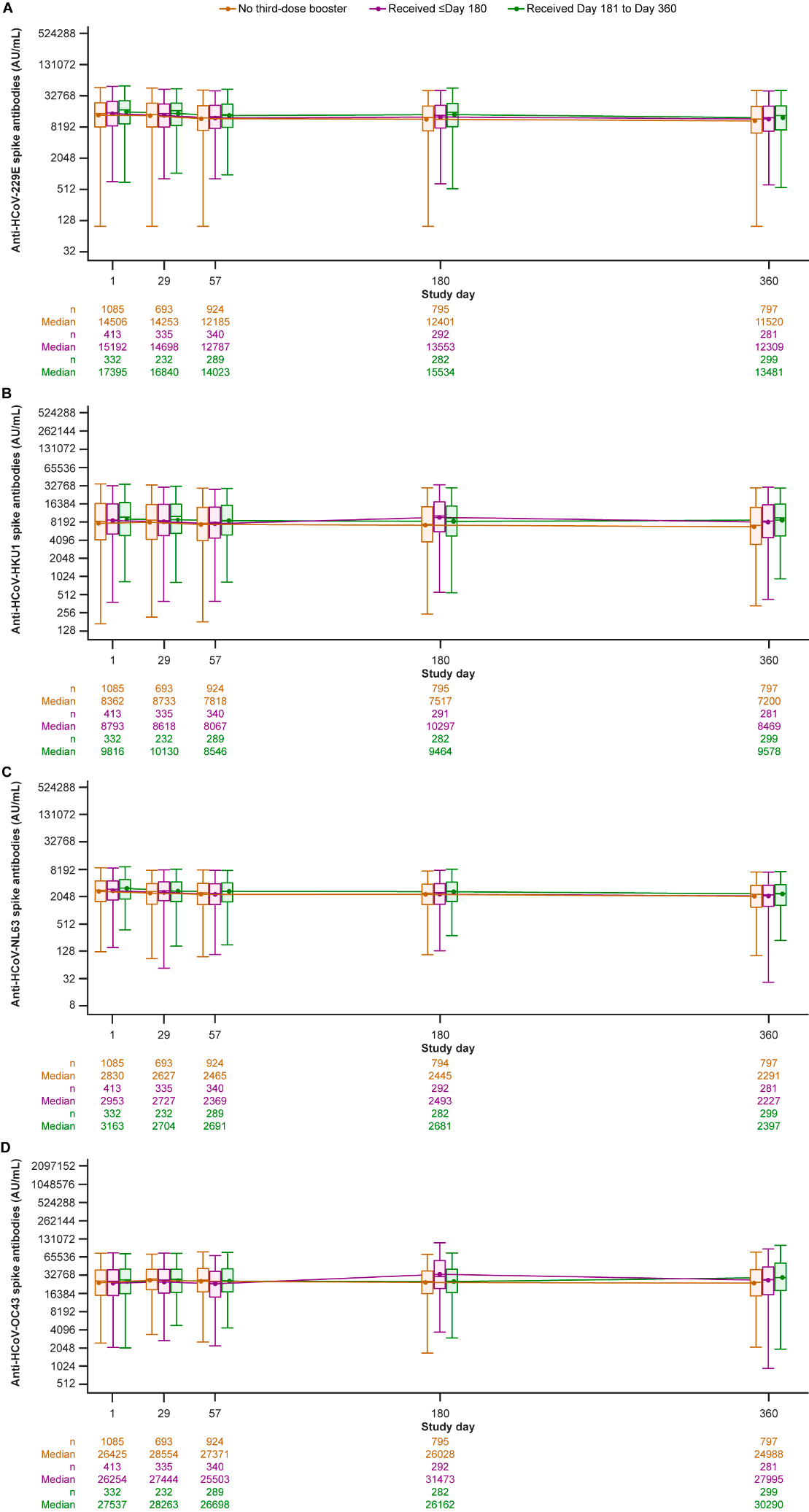
**Supplementary Figure 4. Anti-RSV post-F antibody titers over time in participants who received two doses of AZD1222, by receipt of a third-dose booster.** The bottom and top edges of the box indicate the first and third quartiles (the difference is the IQR) and the line inside the box is the median. The line connects the geometric mean value for every visit. Whiskers extending up to 1.5 times the IQR above the upper quartile and below the lower quartile. Any points more than 1.5 x IQR from the box are considered outliers and are not presented. The boxplots are presented in log2 scale. Baseline is defined as the last non-missing measurement taken prior to the first dose of study intervention (including unscheduled measurements, if any). Titer values measured as below LLoQ are imputed to half the LLoQ. Titer values measured as above ULoQ are imputed at the ULoQ value. Assessments collected after exclusionary restricted medication intake are excluded. The value at Day 1 is the last non-missing value taken prior to the first dose. IQR, interquartile range, LLoQ, lower limit of quantification, post-F, post-fusion F protein, RSV, respiratory syncytial virus, ULoQ, upper limit of quantification.


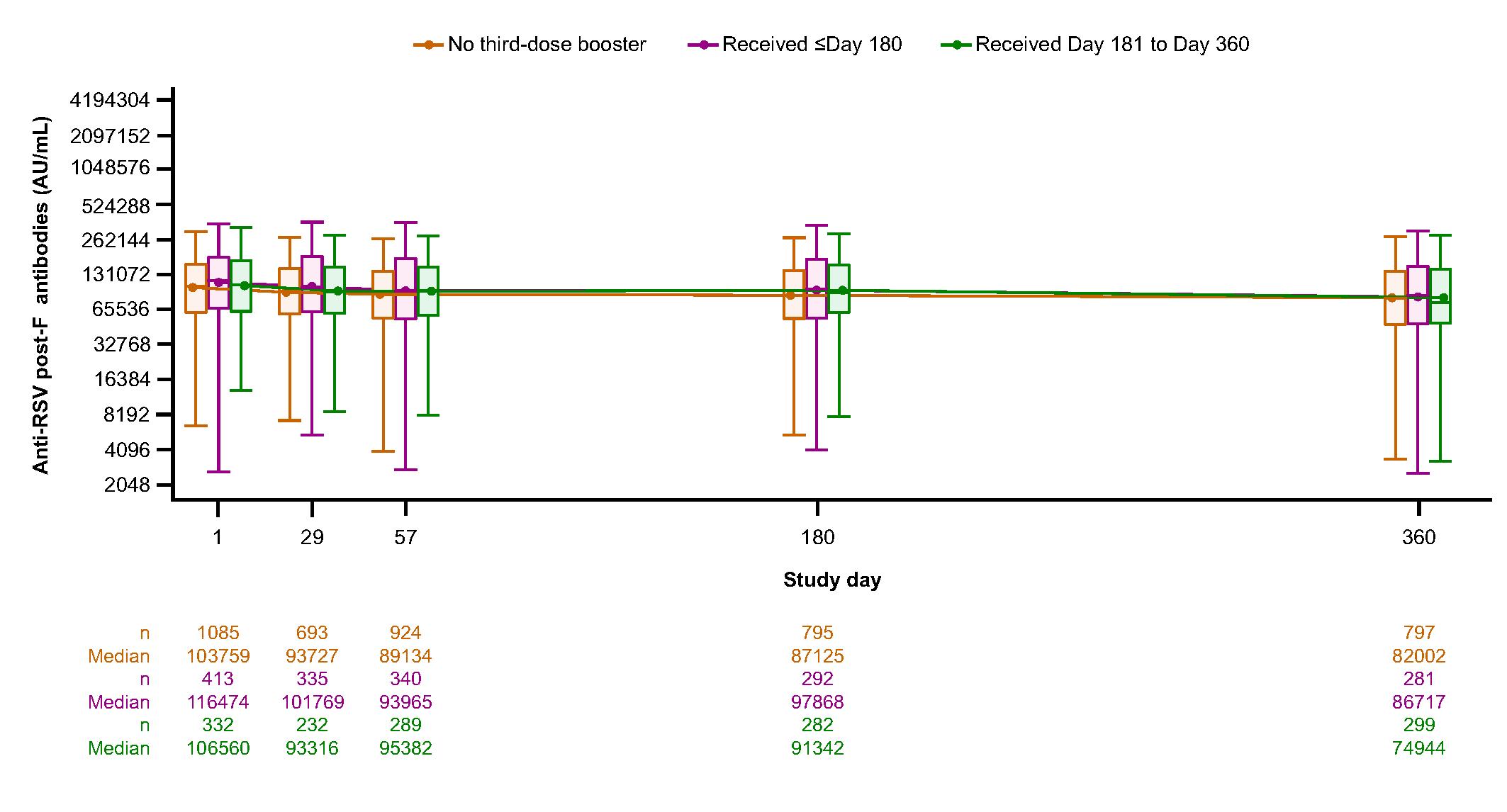


**Supplementary Table 1. Demographics and baseline characteristics of sub-study participants who provided serum samples for the analyses herein.**

| **Characteristic** | **AZD1222 (*n* = 2026)** | **Placebo (*n* = 1009)** | **Total (n = 3035)** |
| --- | --- | --- | --- |
| **Age, mean years (standard deviation)** | 55.0 (15.82) | 54.4 (16.41) | 54.8 (16.02) |
| Median (Min, Max) | 55.0 (18, 100) | 55.0 (18, 90) | 55.0 (18, 100) |
| **Sex, n (%)** | | | |
| Female | 814 (40.2) | 428 (42.4) | 1242 (40.9) |
| Male | 1212 (59.8) | 581 (57.6) | 1793 (59.1) |
| **Ethnicity, n (%)** | | | |
| Not Hispanic or Latino | 1808 (89.2) | 909 (90.1) | 2717 (89.5) |
| Hispanic or Latino | 170 (8.4) | 87 (8.6) | 257 (8.5) |
| Not reported | 43 (2.1) | 13 (1.3) | 56 (1.8) |
| Unknown | 5 (0.2) | 0 | 5 (0.2) |
| **Race, n (%)** | | | |
| White | 1803 (89.0) | 900 (89.2) | 2703 (89.1) |
| Black or African American | 105 (5.2) | 59 (5.8) | 164 (5.4) |
| Asian | 53 (2.6) | 19 (1.9) | 72 (2.4) |
| Multiple^a^ | 26 (1.3) | 11 (1.1) | 37 (1.2) |
| American Indian or Alaskan Native | 13 (0.6) | 10 (1.0) | 23 (0.8) |
| Native Hawaiian or Other Pacific Islander | 3 (0.1) | 1 (<0.1) | 4 (0.1) |
| Unknown | 12 (0.6) | 3 (0.3) | 15 (0.5) |
| Not reported | 11 (0.5) | 6 (0.6) | 17 (0.6) |
| **Serostatus at baseline^b^** | | | |
| Negative | 1983 (97.9) | 973 (96.4) | 2956 (97.4) |
| Positive | 20 (1.0) | 22 (2.2) | 42 (1.4) |
| Missing | 8 (0.4) | 3 (0.3) | 11 (0.4) |
| Not assessed | 15 (0.7) | 11 (1.1) | 26 (0.9) |
| **COVID-19 co-morbidities at baseline^c^** | | | |
| Yes | 1279 (63.1) | 654 (64.8) | 1933 (63.7) |
| No | 747 (36.9) | 355 (35.2) | 1102 (36.3) |

^a^Participants who reported more than one race are reported under 'Multiple'

^b^Serostatus as baseline was defined by above threshold (>9787 AU mL^-1^) nucleocapsid antibody level as per Wilkins et al. (35)

^c^Defined as participants with medical conditions that may place them at a higher risk for acquisition of, or more severe, COVID-19 disease defined as per Falsey et al. (10)

**Supplementary Table 2.** Participants with reported cases of seasonal coronavirus infection, RSV infection, or COVID-19 cases occurring ≥15 Days post second dose (censored at non-study COVID-19 vaccination).

|  | **AZD1222 (N** **= 1896)** | **Placebo (N = 922)** |
| --- | --- | --- |
| **HCoV or RSV infection^a^, n (%)** | 17 (0.9) | 1 (0.1) |
| **HCoV infection^b^, n (%)** |  |  |
| HCoV-229E | 4 (0.2) | 0 |
| HCoV-HKU1 | 1 (0.1) | 0 |
| HCoV- NL63 | 4 (0.2) | 0 |
| HCoV-OC43 | 4 (0.2) | 1 (0.1) |
| **RSV infection^b^, n (%)** | 4 (0.2) | 0 |
| **COVID-19**^c^**, n (%)** | 92 (4.9) | 55 (6.0) |

Participants who received a non-study COVID-19 vaccination prior to 15 days post second dose were excluded from the analysis set. Participants who received a non-study COVID-19 vaccination ≥15 days post second dose were censored at the date of non-study COVID-19 vaccination.

^a^Based on number of participants with any HCoV or RSV infection at a first illness visit

^b^HCoV/RSV infection was determined by RT-PCR positivity on the Biofire^®^ Respiratory Panel 2.0

^c^COVID-19 reported cases included any RT-PCR positive result.

HCoV, human coronavirus, RSV, respiratory syncytial virus, RT-PCR, reverse-transcriptase polymerase chain reaction.

**References**

1. Wilkins D, Aksyuk AA, Ruzin A, Tuffy KM, Green T, Greway R, et al. Validation and performance of a multiplex serology assay to quantify antibody responses following SARS-CoV-2 infection or vaccination. *Clin Transl Immunology* (2022) 11(4):e1385. doi: 10.1002/cti2.1385
2. Falsey AR, Sobieszczyk ME, Hirsch I, Sproule S, Robb ML, Corey L, et al. Phase 3 safety and efficacy of AZD1222 (ChAdOx1 nCoV-19) Covid-19 vaccine. *N Engl J Med* (2021) 385(25):2348-60. doi: 10.1056/NEJMoa2105290
